# Understanding the Terminology for Snack Foods and Their Texture by Consumers in Four Languages: A Qualitative Study

**DOI:** 10.3390/foods8100484

**Published:** 2019-10-12

**Authors:** Rajesh Kumar, Edgar Chambers

**Affiliations:** Center for Sensory Analysis and Consumer Behavior, Kansas State University, 1310 Research Park Dr., Ice Hall, Manhattan, KS 66502, USA; krajesh@ksu.edu

**Keywords:** lexicon, texture, consumer, snack, cross-cultural, descriptive, sensory

## Abstract

The choice of food products is affected by the combination of food properties, consumer motives, emotions, and context, especially in cross-cultural studies. The designs of cross-cultural studies involve several limitations such as conceptual perception and linguistic and cultural differences in response style. These factors confine the validity and generalizability of such study models. In this study, we have combined linguistic and contextual perception together to generate consumer texture terminologies. Four focus groups discussions were conducted with consumers from nine different countries in English, Hindi, Mandarin, and Spanish. Vocabularies for sixteen texture terms were generated. Consumers provided a single consensus term that they typically use to describe contextual sensory perception. The results show that consumers use several terms to describe texture, and terms are very specific to product and related perception. The English translation of words like “snack”, “texture”, and other sensory texture terms are meaningless for non-English speaking cultures. Researchers are encouraged to validate (test) the structure of cross-cultural study models before application. Practical application: The findings of this study present a model which can be utilized to conduct cross-cultural research studies. The results can contribute to generate accurate consumer responses, acceptance, preference, and addressing consumers concerns. Food industries could leverage these by using our methodology in product development, finding consumer insights, effective communication, and products testing in international settings.

## 1. Introduction

Cross-cultural understating of sensory terminologies is a major need of today’s global world where the same products are tested and marketed internationally. The growing demand for standards to describe products on a global scale makes it more important to define and understand sensory terminologies, either in analytical sensory description with trained panelists or with consumers to investigate human perception [1,2]. Sensory profiling can help to achieve better understanding of products and meet objectives [3]. However, cross-cultural sensory studies become complicated when understanding food perceptions. Issues such as language and culture can promote frustration when trying to understand the same products across multiple countries. For descriptive sensory analysis, such problems can be overcome by training and good communication among researchers and panels [4]. This may be less easy to do with consumers, who may have high variability in their use of consumer terms, a problem aggravated by differences in language and culture. For effective communication across cultures, particularly when consumers are involved, it is vital to understand how people of different languages and cultures describe the same perception.

Texture is an important multi-parameter sensory property stimulating consumers’ attitudes towards foods [5]. In some products, texture is more important than flavor [6]. It is essential to comprehend the structure of texture vocabulary (terms) from the consumers’ point of view, instead of just simply translating them into other languages. Exploring appropriate consumers’ texture terms, describing particular texture perceptions of the consumers in daily life, can help to: (a) better design food products to meet specific needs; (b) address consumer texture concerns; (c) avoid misunderstanding that can occur from simple translations; (d) accurately measure sensory meaning of consumer perception; and (e) help promote marketing that directly speaks to consumer needs. Szczesniak and other researchers gave importance to developing texture lexicons and classifying texture terms in various languages [6,7]. Since those early days of texture studies, translations and comparisons of texture terms among different languages have been an important topic for research. Nevertheless, languages contain many nuances in words, and the topic can quickly become complicated.

Drake [8] developed a list of 54 English texture terms and had approximately 50 English proficient collaborators with texture expertise to translate those terms into 22 other languages. The results indicated that some languages use a single word for multiple texture attributes (for example, *katai* in Japanese corresponds to rigid, stiff, hard, firm, or tough in English). Although the English terms were described by distinguishable terms in another language, the author concluded that translations might result in misunderstanding and inconsistencies because English words were presented out of context. The other drawback was the exclusion of consumers, and the use of highly qualified sensory expertise completely differs from consumers in contextual textural perception. For example, one paper [9] had an English panel to generate descriptors for chocolate, which were then translated into Norwegian and used by a panel. The panels used attribute “fruity” differently. The authors concluded that the fundamental perceptual dimensions were similar across cultures, but the underlying sensory dimension and vocabulary differed.

The problem in simply translating terms was highlighted in a study comparing English and Finnish texture terms [2]. Because terms can have multiple meanings, inconsistency can arise. The researchers provided pre-selected texture terms to consumers, which might have restricted consumers’ own vocabularies. In addition, food samples were not provided for textural experience. Several other studies [10,11,12,13,14] emphasized the differences in the use of textural terms among cultures. The majority of studies either compared existing texture vocabularies or used direct translation of terms into different languages without consideration of specific products or the nuances that exist among languages. However, the general consensus was that the major dimensions of texture vocabulary are consistent across cultures and languages.

Some studies have compared texture vocabularies for specific foods among different languages. French and Vietnamese panels individually generated and defined a set of texture descriptors to profile jellies [15]. The lexicons that were developed were then assessed against preselected sensory descriptors, which allowed successful translation and transfer of attributes to panels in their respective countries. Son et al. [16] used cooked rice as a model product to develop a lexicon to describe rice texture in four countries, i.e., France, Japan, Korea, and Thailand. Lists of terms were generated by naïve panels, and the authors noted that the wealth of vocabulary for texture and aroma was influenced by culture. The most texture terms were generated by Thai panelists, but all terms were semantically similar when translated into English. Zannoni [17] highlighted that while translating texture terms, it is essential to focus mainly on stimuli rather than on words. These results established that direct translation of texture terms isolated from their context could be very problematic. Hence, it can be hypothesized that the consumer understanding of texture is strongly related to the sensory perception experience.

One way to better understand consumer terminology is through focus groups. Focus groups typically involve a roundtable discussion centered on particular issues. The groups must be led by qualified moderators. Focus groups are best suited for clarification of problems, consumer perspectives, attitudes, reactions, motivations, and emotions [18,19,20]. A “laddering” probing style that leads to deeper understanding of the reasons behind participants’ responses or comments can be used to provide depth of information [21]. The focus group method is a unique method to capture significant sensory information that could be otherwise missed [22,23]. Focus groups have been used successfully to generate consumer descriptive sensory terms for mung beans [24], mayonnaise [25], pudding [26], and peanut butter [27]. Qualitative methodology is a well-practiced technique to explore consumers’ knowledge systems, vocabularies, beliefs, and the phraseology that they use to talk about foods.

The overall objective of this study was to determine consumer terminology that corresponds to descriptive sensory terminology for selected characteristics of snack food texture in four languages: English, Mandarin (Chinese), Spanish, and Hindi. Specific objectives of this study were (1) to obtain a consumer meaningful texture vocabulary for key aspects of snack foods, (2) provide positive and negative connotations associated with texture vocabulary, and (3) determine whether simple translations of sensory terms to consumer language would be appropriate. Additional information on the role that snacks play was collected too.

## 2. Materials and Methods

### 2.1. Participant Profile

This study was conducted at the Center for Sensory Analysis, Kansas State University, Manhattan, KS, USA. The city is a hub of international communities living, working, and studying at Kansas State University. It also has a substantial population of military family, many with spouses from foreign countries, and immigrants who have settled in the multicultural community. The consumers were recruited via an established database of community participants using an online screener with predetermined quotas. To qualify for the study, all consumers had to eat snacks at least once a week, have no food allergies or dietary restrictions, and could not have an educational background in food/nutrition, dairy, or sensory sciences. Participants for the focus groups in the specific languages (Hindi, Mandarin, and Spanish) had to be a native speaker of the language and had to have been living in the United States (US) of America for less than two years. All non-US participants also had to have a basic understanding of English, but fluency was not required. US consumers had to be native English speakers and have lived in the US more than 10 years. Hindi-speaking consumers were residents of India. Mandarin speaking consumers were residents of China. Spanish-speaking consumers were from Mexico, Costa Rica, Argentina, Ecuador, Colombia, and Uruguay. Female participants were at least 50% or more for each group (Table 1).

### 2.2. Products

The list of representative foods served to consumers to establish textural context and to help in determining consumer term options was based on the descriptive sensory analysis results produced by Kumar and Chambers [1], who used a trained panel and expert translators to describe textural terms in various languages (Table 2). The samples used in the study were ready to eat without any preparation and, thus, were served “as is”. The samples were served blind (no label information) in 3.25 oz (plastic) or 8 oz (Styrofoam) cups (based on the size and shape of the samples) and covered with a lid. One sample at a time was served to consumers for tasting. Participants cleaned their palates between samples with water. Paper napkins were provided for cleaning of lips and hands.

### 2.3. Focus Group Methodology

Professionally trained moderators, whose native language was that of the consumer group, led and conducted four focus group discussions. The moderator’s guide was prepared in English (Table 3) and moved from more general to complex, and on to detailed questions. After discussions with industry colleagues, it was translated into three languages by the moderators, who also had excellent skills in English. Whenever a question arose about possible options for translation, other native speakers of that language were consulted. All sessions were conducted in the native language of the representative consumer group, and were video recorded in order to review later. Each focus group session lasted for 90 min. The study was approved by the Committee on Research with Human Subjects at Kansas State University.

Each participant was provided with printed handouts that included the trained descriptive panel texture terms of interest (Table 3) with definitions in both the native language and English [1]. The terms and definitions were provided one at a time, at the time the term was discussed. To provide context, participants were served representative snack foods for each attribute listed in Table 3. Recent authors have shown that simple changes in the flavor of products where texture is maintained can still show differences in consumer emotional response to the product, clearly indicating the importance of tasting to provide context [28].

The participants were asked to read the textural term and definition, followed by tasting of product for textural experience. Then, participants were asked to describe the textural attribute using consumer terms in their native language that they thought were best representative of the attribute, definition, and the experience during tasting. Multiple terms were requested from the participants; the moderator obtained at least three terms before exploring the meanings of those words and discussing them. Once the list was developed, discussion on the meanings of those terms and how they compared were held to begin developing consensus for the one best consumer term, if possible, that was most representative of the descriptive texture term developed by a trained panel. The strategy was to get the most appropriate term that consumers usually use to define these textures in their daily life.

## 3. Results

### 3.1. Snacks and Texture

The basic concept of the term “snack food” was the same among participants from all countries: Convenient, something that is small, quick, packaged ready to eat, eaten between meals, and not considered healthy. While enquiring about the terminology’s consumer use for snack and snack-like foods, we found that no specific word or term exists for snacks in the Spanish, Mandarin, and Hindi languages. Also, no translation terms exist for “snacks” in these languages. However, consumers used product names and/or some related terms. For example, the Chinese group used terms like *“passing time, tasty food, and potato chips*”. Indian consumers used terms such as *nashta* (evening breakfast), *namkeen* (trail mix), *time pass, and alpahar* (small amount of food) for snacks. Out of seven, five Hindi speakers voted for the term “*namkeen”* and two voted for the term “*alpahar”* for snack food in Hindi. The Spanish speakers used a plethora of terms, such as *aperitivo* (side dish), *colación* (a meal that is considerably smaller in calorific content than lunch or dinner), *refrigerio* (snacks, Central America, usually served in meetings/formal gatherings), *picada* (a snack in Argentina consisting of cheeses and usually cured meats), *merienda* (snack), *botana* (a snack usually for parties in Mexico), *piqueo* (snacks, South America), *tapas* (appetizer or snack in Spain), and *bocas* (snack for parties only, Central America). All of the terms used were different and specific to culture, country, occasion, and kind of snack.

There were some differences among groups; for example, the English and Spanish speakers considered snack foods to be something in between meals but not necessarily a meal replacement. In contrast, Hindi and Mandarin speakers suggested that snacks could be used in place of a meal. While defining snack foods, Indian consumers used product names as identifiers, such as potato chips, nuts, and *namkeen* (Hindi name for “trail” mixes), and sensory attributes such as fried, crispy, groundnuts, chocolaty, etc. Snacks have been identified and defined by other researchers based on eating occasion [29,30,31,32,33], type of food [34], amount of food consumed, location of food consumption, or a combination of several of these factors [31,35,36,37]. Phan and Chambers [38,39] had consumers identify snack foods based on morning, afternoon, and late night eating, and then determined the types of snacks consumers ate during each of those occasions. Breakfast cereals, dairy, egg products, and baked products were preferred in the morning. Fruit, nut, and seed snacks were mostly consumed during mid-morning snacking compared to any other occasion. Legumes and legume-based products were for mid-afternoon and late-night snacking. Sweets were mainly consumed as late-night snacks [39]. Phan and Chambers [38] also reported snacking as indulgent and part of daily meals among US consumers.

Participants related snack food purchase and consumption to liking and their cultural background and previous experiences. Cost, package size, packaging (attractiveness and information), nutrition, calories, labeling, and brand names were mentioned as common factors among groups. The other important aspects that influence snacks purchase were health, fat content, calories, protein content, emotions, family members, and resealability. Similar motivation, such as liking, convenience, energy need, hunger, and health, were reported as primary drivers for snacking among US consumers by Phan and Chambers [38]. Only English speakers (US group) mentioned texture as a driving factor for snack purchase. Other consumer groups did not mention texture explicitly. However, the terms used by these groups, such as fried, crispy, and crunchy, were texture terms, but consumers did not associate them with texture or failed to relate these terms as texture properties of snack foods. Hindi and Spanish speakers also talked about flavor (taste) attributes—for example, sweet, spicy, and salty.

We found no specific word or terminology for the term “texture” in Spanish, Mandarin, or Hindi—and no translation terms exist for “texture” in these languages. Consumers used both terms and phrases to define texture, and some of these terms cannot be translated well into English. The three terms provided by Hind speakers were *sanrachna* (structure), *upari parat* (upper layer), and *haath se chuu kar pata lagana* (hand feel). Consumers rarely use these terms because they are complex and uncommon in the culture. The Mandarin Chinese group gave the terms 口感 (mouth feel or how food feels in the mouth) and 触感 (hand feel).

The English speakers from the US were the only group that related sensory perceptions to the term “texture” and explained “texture” explicitly as a terminology. For example, “*a crunchy texture of an apple is an indicator of freshness, whereas mushy apple is stale”, “I do not eat crunchy textured foods because it’s noisy*”, and *“yogurt is too thin to experience, not my jam, rather I like hummus because it is more substantial”*. Hindi, Mandarin, and Spanish speakers had difficulty with “texture” as a translated term and used different methods to describe what “texture” was.

General comments made by Hindi speakers on snack texture were “*ruffle texture, structure of food, rough surface, crispy, and indentions on chips*”. They were not able to understand the English term “texture” as a terminology for snacks, and their responses were mainly based on individual experiences. Some direct comments were “*hardness depends on chip, kettle cooked is different from normal chips”,* said a 26-year-old women. Indian consumers frequently used product names and associated sensory attributes such as namkeen mixture, bhujia, roasted groundnuts, bhel puri, salty, spicy, crispy, crunchy, sweet etc. Similarly, Chinese speakers frequently used product names to establish textural concepts in terms of specific products. For example, *“peanut candy should be crispy but not hard, and I will be disappointed if it is very hard”, “softness of bread, it should not be dry or hard”, “creaminess and thickness of yogurt texture”, “liquid texture of yogurt stimulates the feeling of low quality”* etc. “*A cracker needs to be crunchy*”, said a 32-year-old Spanish speaker. The Spanish-speaking group related textures to snacking occasion. For example, *“what texture I eat depends on the time of the day and the event*” and *“cereal bars for the office consumption”*. A few Spanish speakers noted that texture was a quality, freshness, and purity indicator. For example, “*fruits with certain textures are too perfect, it makes me think if it has something extra”*.

All groups had experienced multi-textured snack foods before, but not all were as adept at describing the textural aspects of such products. For example, Hindi speakers often attributed their prior experience to multi-sensory characteristics, including flavor (i.e., taste and aroma). For example, *“rasagulla (sweet dumpling) liked for sponge feel, sweet taste and rose flavor”,* a product *“layered in cookies and ladoos (sweet dish) coated with coconut flakes offers a variety of soft and hard bite experiences”,* said a 26-year-old woman. Other examples provided for multi-textured experiences were “*ice cream as soft, apple as crispy, and banana as soft*”, suggesting again that the concept of the term “texture” was associated with specific products. Spanish speakers talked about combining different snacks with alcoholic “beer” and non-alcoholic “coffee and tea” beverages. For example, *“flour or grain-based snacks pair well with coffee or tea”* and “*meat snacks go well with alcohol*”. The US group shared widespread multi-textured experiences such as *“soft yogurt with crunchy granola”*. Some snacks were preferred for their specific features, such as *“crispy and flaky pretzel stick for their crunch and thick bite experience” and “buffalo pretzels for crispness”*.

### 3.2. Snacking Occasion, Texture, and Emotions

We found a strong association between snacking occasion, textures, and emotions. The format of association remained consistent across cultures and genders. Consumers want to start their day with a soft-textured snack or food, and as the day progressed, consumers tend to move towards more crispy, hard, and noisy textures. In addition, consumers prefer to eat something healthier or something close to nature, like fruits, particularly in the morning and early in the day. Consumers want to avoid noisy snacks in public places. For example, *“in office, I don’t like crispy stuff because it is super loud”, “I prefer soft texture like chocolate or fruits like banana. I have issue with apple because it is still noisy to eat”,* said a 24-year-old Indian woman. “*Usually I eat fruits in morning, it is culture to eat fruits in morning and savory snack in evening”,* said a 26-year-old Indian woman. Similar responses were received from the Chinese- and Spanish-speaking groups.

Consumers associated occasions to snack eating, for example *“sometimes I need sweet in the morning more like fruit or other sweet, and in the afternoon more crunchy or salty*”, said a Spanish speaker. A pattern of starting the day with something sweet, soft, less or no crisp or crunch at all was also observed among non-English speakers. The Indian group preferred to eat fruits, milk, puff pastry, croissants, bread, and soft-textured foods, giving the reason as tradition and culture. Some specific comments included *“I eat soft textured food in morning, even if I eat rusk (sweet toast) like food, I dip that in milk or tea to make it soft, little hard during lunch but likes crispy in evening”,* said a 26-year-old women. *“I do not like to eat spicy or savory snack in morning, prefer to eat something that gives me a less stomach fill experience”,* said 30-year-old man. The majority of Indian consumers eat savory, crispy, crunchy, and hard-textured snacks in the evening or late at night to re-energize themselves. At night, they prefer to eat sweet, semi-liquid snacks like chocolate. A 30-year-old Indian consumer said *“I want to eat something in night where I do not waste energy to chew or eat”*. One or two consumers in each group did not have a texture preference at all. The number of consumers who had “no texture preference at all” was higher in US group than others, but still in the minority.

Consumers mentioned some specific textures that are preferred at certain places. For example, all groups want to avoid eating crispy and crunchy textures in public (like offices) due to noise generation. For example, *“In office, I don’t like crispy stuff because it is super loud, I prefer soft texture like chocolate, fruits like banana, have issue with apple too because it is still noisy”,* said a 25-year-old Indian woman. A 28-year-old US woman said *“I don’t like to eat crumbly, easily breakable, sticky, and oily snacks in the car”*. Chinese consumers also shared similar comments about avoiding eating snacks in public places that makes noise. Spanish consumers prefer to eat creamy textures at home due to spreading process. US consumers usually eat snacks in all places, but mostly prefer to eat only at home. Alike, India consumers mostly eat snacks at home.

We found a strong association of snacking with emotions. When stressed, some consumers prefer to snack, whereas others said they lose their appetite. Consumers, especially women, prefer to eat sweet and soft-textured snacks (like ice creams and cookies) when feeling sad. Specific comments made by the US group were *“if it’s a bad day, that’s an excuse to get ice cream or cookies”, “when frustrated I eat crunchy and loud to get steam out”, “if sad, eat ice cream”, “If sad or down, I eat ice cream”*, and *“if stressed, I do not eat anything”*. In the comments, “taste” (such as sweet) dominated over texture preference for most emotions, at least among women. For example, a 34-year-old US woman in the English group stated *“my snacking preference in a sad emotional state is more related to taste and flavor than texture. I do not eat savory foods when feeling sad”*.

A contrasting pattern was found for male consumers. Almost all male consumers either eat anything (texture does not matter) or prefer to eat crunchy, savory, and hard-textured snacks when they feel angry, saying it helps to get the anger out, and potentially exhaust themselves. US consumers were found very assertive for their emotions and snacking behavior, whereas Spanish-, Chinese-, and Indian-speaking consumers were more assertive for occasion (different time in a day), places, and snacking behaviors than emotional associations.

### 3.3. Consumer Texture Terms

Table 4 (English), Table 5 (Hindi), Table 6 (Spanish), and Table 7 (Mandarin) represent all consumers’ descriptors used for each texture attribute and final terms (Table 8) on which each group agreed. We used the closest English meaning of each term to explain results. Original terms in native languages can be found in Table 4, Table 5, Table 6 and Table 7.

Gummy Worms were used as a reference food for “firmness”. US consumers described *“force required to bite completely through the food sample with the molar teeth”* as chewiness, toughness, and hardness. The Hindi-speaking group used exactly the same terms, the Chinese group used chewy and toughness, and the Spanish group described firmness as consistency, hardness, and resistance. Only “hardness” was consistent among all four groups. The US and Indian groups described “toughness” as the most suitable descriptor for firmness, based on the (1) tough structure of the Gummy Worms and (2) the force required to bite through the Gummy worms. The Spanish and Chinese groups preferred terms that translated as resistance and chewy as their final descriptors (Table 8). The reason was the high number of chews required to breakdown the food.

Brach’s chocolate balls were used as a reference food for “smoothness”. The descriptors used by US consumers were sleek, creamy, silky, clean, clear, hard surface, and smoothness. The group explained that the terms might change with product. For example, “*creamy goes for cheese spreads and silky for milk-based drinks*”. The Indian group used six terms to describe smoothness, but not all terms could be used in a similar manner. The terms were gluey, slickness, soft feel (कोमल), slippery/smooth (चिकना), soft feel (नरम), and soft touch (मृदु). The terms largely mean smoothness, but terms were specific to certain products. Spanish speakers used flat, smooth (terso), soft, smooth (liso), and homogeneous. There is no term for smoothness (only smooth) in the Spanish language. The Chinese group used roundness and smooth. Only smooth/smoothness was common among cultures. Due to the smooth surface of chocolate balls, all groups felt smooth was the most suitable final term (Table 8). The final terms may change to other terms if the product is different. For example, Chinese consumers used “smoothness” for hard-textured products and “roundness” for soft-textured products.

Frozen jackfruit was used as a reference product for “moistness”. US consumers used wet, slimy, juicy, and tender as descriptors and stated that they use tender for moist meat food and slimy for oyster-like watery foods. Indian consumers used descriptors such as water-like (पानी पानी), water-like (पनियाल), flabby, भेजवाला (no English term), and wet. Spanish speakers used juiciness, watery, and wet. The Chinese group used only two terms, i.e., juicy and moist (wet). The term “wet” was common among the four cultures, and “juicy” was common in three of cultures but not in the Indian group. The term “juicy” in Hindi explicitly reflects fruit or vegetable juice perception. Therefore, Indian consumers used “wet” as a term for moist perception of jackfruit. Whereas, other cultures used “juiciness/juicy” for dripping-moistened jackfruit. The perception of “juiciness” as a fruit juice, not as moist, was the dominant driving force here. Again, the final terms may change if the product is changed.

Sourdough pretzels were used as a reference product for “roughness of surface”. US consumers used coarse, abrasive, gritty, and jagged as descriptors, while the Hindi-speaking group used hard, rough/abrasive, and crisp. The Spanish group used six descriptors, i.e., roughness (rugosidad), heterogeneous surface, irregular surface, rough, lijoso (sharp-edged products), and roughness (aspereza). The Chinese group used rugged and roughness, with “rugged” as the final term (Table 8). The Spanish and US consumers settled with “rough” as the final term. Indian consumers determined that both rough and abrasive were equally good (Table 8). The rough perception was due to the top surface of the pretzels. Both the US and Indian consumers may use other terms for “roughness of surface” if the product is different.

For “adhesive” perception, chewy caramels were used as a reference product. US consumers used descriptors such as sticky, chewy, gummy, and tacky. Indian consumers used sticky (चिपचिपा), sticky (चिपकना), and slippery (चिकना). Spanish consumers used sticky, taffy, and gummy, and Chinese consumers used teeth sticky and viscous. The US consumers commented that “the *product is chewy, and it sticks on teeth because it is chewy*”, and chose “chewy” as the final term. The other three cultures used “sticky” (teeth sticky) as final term (Table 8). “Adhesive” was too technical, as consumers use “adhesive” to describe adhesive glues for pasting things but not as a food sensory descriptor.

US consumers described “cohesiveness” by terms such as chewy, gummy, crumbly, spongy, uniform bite, and “change in shape but stay as whole”. There was no agreement on the use of a single term, so no conclusion was reached. Indian consumers used strong, tough, “something that holds together” (न टूटने वाला), “remains together” (जुड़ा हुआ), “something that does not break”, and “something that does not scatter”. They agreed to use the phrase “something that does not scatter” as the final term. Spanish consumers used terms such as elasticity, softness, consistency, firmness, and brittle; the final term was “elasticity” (Table 8). The Chinese used only one term, “tenacity” (something that does not break or recover in shape, like sponge). Cohesiveness is a complex term that encompasses multiple aspects.

The US consumers described “crunchiness” as crunchy and crackly. Spanish consumers used crunchiness (crujencia) and crunchiness (crocancia), with “crunchiness” (crocancia) as the final term. Similarly, Indian consumers also used crispy as the final term. Hindi speakers used only one Hindi term to describe both crispy and crunchy, i.e., कुरकुरा (Kurkura, it does not translate well into English). Chinese consumers used descriptors crispy and crunchy, with “crispy” as the final term (Table 8).

Lay’s classic potato chips were used as a reference product for “uniformity of bite”. The US consumers used descriptors such as consistency of texture, hardness, brittle, one bite, disintegrate, smooth, and consistency (of bite); the final consensus term was “consistency of bite” (Table 4). Indian consumers used light, easy to break (bite), uniformity of bite, and easy to bite; the final term chosen was “easy to bite”, although this may not be exactly the same concept. Spanish consumers used descriptors like homogeneity of bite, uniformity, consistency of bite, and resistance of bite; the final terms agreed was “homogeneity of bite”. Chinese consumer used a single term, “even texture”, to describe “uniformity of bite”. The consumers associated the perception as “the way a product breaks inside mouth”, which was described as “evenness of bite”.

Oiliness was measured on Lay’s classic potato chips. Both “oily” and “greasy” were used as the final terms (Table 4). The Indian group provided the additional term “fried” for oiliness, but it is generally used as an identifier for fried foods. The moderators enquired to know if consumers perceive oily, greasy, and waxy as the same or different. Spanish consumers used waxy and greasy interchangeably, observing only a small difference that they could not explain. O’Mahony and Alba [40] found inconsistencies among Spanish and English speakers in their choice of descriptive terms for sour/acid or bitter foods. “Oiliness (aceitoso) is more related to the surface properties, and more appropriate for snacks”, explained Indian and US consumers. The consumers understand oily, waxy, and greasy as different, and provided examples to back their opinions: “*Waxy is like a coat to cover a product or coating on skin, and waxy is thick and hard, waxy does not drip, and does not leave any residue on fingers”*. Examples mentioned were *“fruit covered with wax (apple skin), “Laffy Taffy” (a brand of thick hard chewy candy), layer of cheese, and solid state of butter is waxy like pastry dressing”*. *“Greasy is liquid, might drip, and leaves residue on fingers”. “Greasy is like ghee (milk fat), molten state which sticks in mouth, has an after taste, and stays in mouth even after swallowing”*. Examples were *“greasy hot cheese dripping on pizza that comes up on a napkin”,* and *“Suji ka Halwa”* (sweet dish made from semolina and *ghee*). Oily was defined as a thin layer of oil observed on the surface of foods. Overall, the consumer experience of greasy, oily, and waxy was mainly of visual and tactile perception.

Yoplait strawberry yogurt was used as a reference product for “astringency” perception. Terminologies used by US consumers were dry, tabasco, chaps your lips, bitter, sour, salty, spicy, thirsty, and thick. Taste attribute sensations dominated the perception, which may be common with astringency perception of yogurt. Astringency is referred to in the sensory literature both as a part of flavor (trigeminal sensation) and as a texture. Indian consumers used dryness, and Chinese consumers used dry and numbing as descriptors. The Spanish group used astringency as the final term, and the additional terms were roughness and dryness. None of the consumers felt astringency (drying/puckering) in the sample food, whereas a trained descriptive panel found very high intensity of astringency in Yoplait strawberry yogurt [1]. The possible reason could be that the untrained profile of consumers failed to identify the astringency sensation.

For “chew count”, all four cultures used chewy (high number of chews required to breakdown food) as the final term (Table 8). Other terminologies, for example: US consumers used gummy; Spanish consumers used chew-ability; and Indian consumers used “ease of swallowing”. As for Indian consumers, “if something is chewy that it is not easy to swallow”.

US consumers described “residuals in mouth” as gritty and grainy, and Chinese consumers used residuals (indicated the need to drink water to clear teeth). Spanish consumers’ use of terms was based on the lingering sensation, i.e., flavor in the mouth, sensation in the mouth, and residuals in the mouth. Similarly, Indian consumers also used phrases such as “left over in mouth” and “stuck in between teeth”. The US consumers concentrated on the nature of the food, i.e., “gritty”, and other cultures selected “leftover in mouth” as the final term (Table 8).

The Spanish consumers described the term “powdery” as “dusty”, but other cultures used “powdery” as the final term (Table 8). Multiple terminologies were provided by each group of language speakers, with US consumers using chalky and dry; Indians using flour-like; and Spanish using sandy, grainy, floury, dusty (Polvoso), and dusty (Polvoroso). Although powdery is an English term, all four cultures understood it fairly well.

Consumers found it difficult to relate to “dissolvability” as a term for food texture sensory perception. The US consumers used terms such as disintegrate, airy, melt, and dissolve; Indian consumers used melt and dissolve; Spanish consumers used solubility (melt) and dissolvability; and Chinese consumers used a single term, “dissolve”, as the descriptor. All others groups preferred to use “melt” as a generic texture term for “dissolvability”.

Heat burn was noted by consumers to be more of a taste sensation rather than texture. The terms used by US consumers were spicy, hot, real hot, hot-hot (for Spanish foods), flaming, lips burning, chili powder, and chili pepper. Indian consumers used peppery hot, spicy, and peppery tang; Spanish consumers used spicy (picante), spicy (picosidad), and spicy (enchiloso); Chinese consumers used burning, spicy, and pungent. The terms spicy (picante) (used by both Spanish and US consumers), peppery hot (Indian consumers) and burning (Chinese consumers) were used as the final terms (Table 8).

A crunchy granola bar was used as a reference product for “particle amount”. The terms generated were crumbly, grainy, and gritty (by US consumers); crisp, gritty, and crumbly (by Indian consumers); grainy (by Spanish consumers); and granular and crisp (by Chinese consumers). The final terms were grainy (by both Spanish- and English-speaking consumers), crumbly (by Indian consumers), and granular (by Chinese consumers) (Table 8).

## 4. Discussion

Consumers related texture with quality, freshness, taste, ease of handling, and good experience. Consumers had certain texture benchmark expectations for each snack food, which must be met for acceptance of that particular snack food. The consumer benchmark expectations were completely based on experiences from previous consumption of those snack foods. For example, a benchmark for chips (crisps) is that there must be a certain level of crispness without the chip being either limp or too hard, and the chip must shatter without being powdery or breaking into pieces with sharp edges. Consumers had positive and negative connotations with textures. Consumers considered snacks that are too hard, too floury (starchy), too gummy, or oily as negative textures that discourage them from eating or handling snacks. However, airy and crunchy (indicator of freshness, good quality) are positive textures. For example, *“good texture also tells us about the ingredients used in snack manufacturing, for example, the creaminess of an ice cream”*.

English speakers (US) used more vocabulary to define their understanding of snacks without naming food products. The non-English speakers used food product names to explain their concepts of snacks. The understating of snacks for non-English speakers was mainly context-based. For example, “*potato chips, nuts, namkeen, crackers, candies”* etc. Therefore, sensory studies of non-English speaking cultures conducted on direct English translation without any contextual backing might be misleading. Some authors [13,15,16,17,40,41] emphasized (a) the importance of context in identifying consumers’ sensory descriptors, and (b) that translation of sensory descriptors among different languages are always not useful. Vlontzos et al. [42] used a technique where a questionnaire was developed, translated into two languages, pre-tested in each language representing Eastern and Western European countries, and finally translated into the seven languages used in the test. In contrast, other researchers [43,44,45,46] have used translation and back translation to the original language to confirm that the meaning was maintained.

Spanish, Hindi, and Mandarin do not have a specific term or terms for “snack” or “snack foods”. These languages do not have a direct translation similar to snack or snack foods, although there are context-specific words driven by time of day or eating occasions. The specific sensory terms provided by consumers were different, and the majority of consumers do not use these terms in daily life. However, Chinese and Indian consumers had one commonality in explaining texture that is something perceived by *“handfeel”*. This assertion confirms the basic definition of sensory perception by one of the five senses.

We found that only English speakers explicitly mentioned texture as a factor they consider while making a snack food purchase. Speakers from other languages used terms that represented particular textures of specific foods, such as “crispy apple, crunchy chips” etc. Languages such as Hindi and Mandarin do not have consumer-relevant terms for the overall concept of “texture”. No term, not even translation, for words like “texture” exist in Spanish, Hindi, or Mandarin. The use of the English term “texture” for Indian, Chinese, and Spanish consumers is meaningless. Conceptual differences across cultures resulted in consumers responding somewhat differently. The non-English language groups’ understanding of texture was based on their previous experiences and memories of certain foods, which they often used to support their comments [47]. The consumer experiences and memories of certain food textures can be termed as “contextual experience”, which is one tool to overcome language and understanding barriers in non-English speaking cultures. One paper reported on a technique similar to what we used with determination of attributes by trained panelists, translation, and back translation by experts, and then using representative products and the lexicon to produce a multi-lingual questionnaire for use in various countries [48].

These findings may be applicable to online surveys where sensory questions are presented without contextual references. Eertmans et al. [49] reported the lack of completeness of food choice questionnaire models (FCQ) and their generalizability to a wide range of countries. The authors suggested that the meaning and connotation of the items may be strongly affected by culture. Steptoe et al. [50] emphasized the need to relook and revise questionnaires investigating the perception of consumers from different cultures to include items related to the main factors of their food choices.

All groups had experienced multi-textured snacks before, and everyone in these groups indicated that they enjoyed such snacks. Consumers across cultures preferred to mix foods for balancing texture, flavor, and taste.

A clear association was observed for snacking occasions with specific textures. Mostly, consumers prefer to eat snacks “at home while watching movies, lying down, resting, convenience, relaxing, passing time, studying, when they see snacks at home that they like, do not want to share with others, and do not want to look bad in office”. Phan and Chambers [39] also reported snacking as to be a more personal event. All of the activities mentioned above are convenient and energy-charging snacking. A US woman consumer mentioned a special category, “fuel texture”, which helps “to gain energy from foods like pretzels or jellybeans, but not heavy or thick-textured foods”. Other authors also defined snacks as energy-dense food [51], and consumers reported to eat snacks to fulfill energy needs [38].

The majority of consumers start their day with soft-textured and sweet-tasting snacks. Indian consumers indicated “culture” as the primary reason to eat soft-textured, less savory, and natural foods early in the day. US consumers eat soft, less crunchy, and less savory snacks for soothing experiences. As the day progresses, consumers tend to move towards crunchy textures and savory flavors or indulgent snacks (ice cream for the end of the day when stressed). This is similar to other findings [38,39]. Some emotions were related to snack food textures, although this would need to be confirmed by larger studies. When sad, for example, women prefer to eat soft-textured and sweet-tasting snacks, whereas men tend to eat savory and crunchy snacks when they are angry. Some men commented that taking a snack break when stressed would help to release stress and gain focus.

Results showed wide-ranging perceptions, understandings, preferences, and liking of several textures across cultures. The non-English speakers frequently used taste and aroma descriptors to describe snacks, and texture was seldom mentioned, although their descriptions of food they eat as snacks clearly showed variation in texture. In addition, its relevance was demonstrated by presenting consumers with food samples. Texture has been found to be a strong driver of food liking and aversion, along with flavor [52]. Our texture theme discussion results suggest that consumers (individuals) bring to each food a certain texture expectation. If that expectation is met, then there is less focus on texture. If the expectation is not met, then food is rejected. Our findings on consumer texture expectation of foods are in agreement with Engelen and de Wijk [53]. This expectation of certain texture varies by individual. The variation could be due to a function of consumer prior expectations and experiences for specific foods.

We conclude from these focus groups that the consumers we tested with native languages other than English seem to be less aware of terms similar to those used in English for food texture than English-speaking consumers. In addition, direct translation of texture terms from English into other languages could lead to misleading identifications, if not backed by specific foods for textural contexts. The vocabulary used by non-English consumers to describe sensory perceptions was different among cultures, and was product-specific. Yoshikawa et al. [54] reported that Japanese consumers were more sensitive to subtle variations in texture and had a much richer texture vocabulary than American consumers. Similar, differences in how consumers in different cultures describe sensory attributes have been reported by numerous authors [4,17,40,55].

Although some similarities exist with consumers, no specific consistency was observed in understanding and usage of texture terms among cultures. Hindi speakers used the term *kurkure* to describe both crispy and crunchy experiences, but the term has no direct English translation. Consumers used multiple terms to describe single sensory perception and vice versa. Spanish and Mandarin speakers used grainy and granular to describe roughness of surface, particle amount, and powdery. Similarly, the French term *doux*, which means smoothness and sweetness, has no direct translation into English or Spanish [56]. Consumers found English terms as too technical and confusing, and the direct translations were not always commonly used in a food context. The use of definitions helped consumers in understanding the attributes, but this creates a problem when marketing specific texture concepts to consumers. When marketing to consumers, it is important that the consumer can understand the term. For words where the meaning is not completely obvious, the use of context or other marketing tactics must clearly demonstrate the meaning of the term. Consumers demonstrated similarities in some texture terms, but their conceptual meanings were completely different. For example, the speakers of Hindi and English described “oily” as a surface property of snacks, whereas Spanish speakers used both oily and greasy interchangeably. Spanish speakers related greasy to animal-based products like meat, and measured it as whole (overall).

The results show the necessity to focus primarily on stimuli rather than words when dealing with consumers. For example, English speakers used juiciness to describe moistness of jackfruit but stated that the term might be different if the product was different. A similar trend was noticed for smoothness, roughness of surface, and cohesiveness among English speakers. The different conceptual understating for texture terms was present in all four cultures. The direct translation of texture terms isolated from any context could be problematic with consumers [17]. The selection of the context product should be carefully considered for the development of sensory vocabularies with consumers [16].

We conclude that researchers must avoid direct translation of English words, as they are presented out of context and could potentially lead to misunderstanding, inconsistencies, and confusion. It may not be feasible to develop a comprehensive and complete polyglot list of texture terms across cultures. However, after careful investigation, a limited and common contextual texture vocabulary is possible across languages.

### Limitations

The study results are based on the inputs of a small number of specifically recruited participants, and caution must be considered in generalizing the findings to a larger population. However, it may be completely logical to infer from the theme of this study that consumer vocabulary differs greatly from sensory scientist vocabulary. A simple translation of sensory terms in consumer studies does not reflect true responses. The consumer vocabulary generated in this study can only be used for textural context, not for flavor, aroma, or appearance.

## 5. Conclusions

The accelerated pace of globalization has increased the application of cross-cultural sensory and consumer research [57]. The rapid growth of the internet will continue to foster new opportunities from multiple countries at a much faster, easier, and cheaper rate [58]. Cross-cultural study models based on the assumption of conceptual and linguistic equivalence are problematic. The validity of such models should be tested thoroughly before application. Researchers should avoid imposing constructs and models developed in one culture to other cultures.

Consumers used numerous terms to describe the textural properties of various snack food products. Sometimes, the terms were quite consistent across cultures, suggesting an underlying understanding of the concept. Sometimes, the use of terms was mainly contextual-based, i.e., food or snacks versus other products (e.g., the term for “hardness/firmness” in Chinese depends on whether the person is talking about hardness of steel or hardness of foods), and certain terms were product-specific, as noted. Texture terms developed by trained descriptive panels are easy to translate at a scientific level to produce consistent information across panels, but much too technical for use to describe the products to consumers. We found divergent understanding and usage of English terms in each culture. When conducting consumer studies or communicating benefits of products to consumers, it is essential to pre-test the terminology to ensure that the meaning is conveyed appropriately.

Our results conclude that simple translation of sensory terms without context may be problematic in consumer studies. We provide a method where linguistic differences could be minimized, if backed by contextual perception of sensory terms. The direct translation of descriptors from one language to another does not mean that they are intercepted as conveying the same meaning in both languages. The vocabulary used by consumers to describe sensory characteristics depends on context, culture, and previous exposure to different products. Some of the terminologies are specific to products and may change when the product characteristics or product itself change. Hence, it is important to investigate the cultural mindset, and its implications on food testing.

## Figures and Tables

**Table 1:** Participants’ demographic details.

By Age	English	Hindi	Mandarin	Spanish
18–24	2	3	4	1
25–34	4	4	4	5
35–44	1	1	1	4
45–54	1			
Total	8	8	9	10
**By Gender**				
Female	6	5	7	5
Male	2	3	2	5

**Table 2:** List of food samples served to consumers for each texture attribute.

Sample No.	Descriptive Attribute	Products	Manufacturer
1	Firmness	Gummy Worms	Ferrara Candy Company
2	Smoothness	Brach’s chocolate balls	Ferrara Candy Company
3	Moistness	Frozen jack fruit	Flying Horse
4	Roughness of surface	Sourdough Hard Pretzels	SL Snacks National LLC
5	Adhesive	Werther’s Original chewy caramels	August Storck
6	Cohesiveness	Sourdough Hard Pretzels	SL Snacks National LLC
7	Crispiness	Cheetos Crunchy	Frito-Lay
8	Uniformity of bite	Lay’s Classic Potato Chips	Frito-Lay
9	Astringency	Yoplait original yogurt	General Mills/Sodiaal
10	Oiliness/Oily	Lay’s Classic Potato Chips	Frito-Lay
11	Chew count	Werther’s Original chewy caramels	August Storck
12	Residuals in mouth	Sourdough Hard Pretzels	SL Snacks National LLC
13	Powdery	Mochi roll	Yuki & Love
14	Dissolvability	Jet Puff Original Marshmallows	Kraft foods
15	Heat burn	Seaweed chips	Annie Chun’s
16	Particle amount	Nature Valley—crunchy granola bars	General Mills

**Table 3:** Abbreviated interview guide with introduction and themes covered by the moderator in the focus group discussion.

Section	Interview Guide for Focus Group Sessions
Introduction	Welcome note, guidelines, and purpose Participants introduce themselves
Opening question	How often do you eat snacks in a week?
General question	When you think of snacks, what is the first thing that comes to your mind? What are some of the brands that come to your mind about snacks? What are the things you look for in snack foods to make a purchase? What features make a snack food special from your point of view?
Texture theme	What do you understand by “texture” of snack food? How important is texture for you? What other textures you have experienced so far? What terms do you usually use for snacks or snack-like foods? (This question was asked to Hindi-, Mandarin-, and Spanish-speaking consumers) What terms do you usually use for texture of snacks or snack like foods? (This question was asked to Hindi-, Mandarin-, and Spanish-speaking consumers) Do snacking occasions impact the textures you want? If yes, how? Are oily, waxy, and greasy the same or different in your understanding? (This question was asked to Hindi-, English-, and Spanish-speaking consumers only—it was untranslatable in Mandarin)
Texture attributes	What are words you use to describe these texture terms? ***(1) Firmness:*** *The force required to bite completely through the food sample with the molar teeth.* ***(2) Smoothness:** Degree to which the sample feels smooth and free of lumps/particulates as opposed to lumpy, rough, grainy, gritty, and/or sandy.* ***(3) Moistness:** The perceived amount of moisture in the product.* ***(4) Roughness of surface:** The amount of indentations/bumps and surface abrasions which can be perceived by gently manipulating one piece between the thumb & fingers, lips, palate, and/or tongue.* ***(5) Adhesive:** The degree to which the product sticks to the hands or mouth.* ***(6) Cohesiveness:** The degree to which the sample deforms prior to breaking apart when compressed once between the molar teeth.* ***(7) Crispiness:** The intensity of audible sound when the sample is compressed between the molar teeth.* ***(8) Uniformity of bite:** Degree to which the product changes from start to finish in the bite. If the force necessary to bite through the sample changes during the bite, the product is non-uniform. The more consistent the force, the more uniform.* ***(9) Astringency:** Drying sensation on the surface and/or edges of the lips, tongue, and mouth.* ***(10) Oiliness:** The appearance of a fat or oily coating on the surface of the product.* ***(11) Chew count:** Number of chews required to hydrate sample and bring to a state ready to swallow.* ***(12) Residuals in mouth:** Sample remaining in or on surfaces of the mouth after swallowing.* ***(13) Powdery:** A measure of the dry, powdery sensation in hand or mouth.* ***(14) Dissolvability:** Rate and degree to which product dissolves in the mouth during mastication.* ***(15) Heat burn:** Burning sensation on the lips, in the oral cavity, and in the throat, resulting from exposure to a substance such as capsaicin or hot peppers. The sensation tends to persist after the stimulus is removed.* ***(16) Particle amount:** The perception of small particles relatively bigger than surrounding product.*
Closure	When and where do you often eat your snack food?
Additional groups questions	**Only to English-speaking American consumers** Do you think emotions have anything to do with snack eating? How would you design a snack food if provided an opportunity? **Only to Hindi-speaking Indian consumers** People who eat snacks at home. Why do you eat snacks at home?

**Table 4:** Consumer texture terms provided by the English-speaking group (US).

Descriptive Attributes	Consumer Terms	Final Consumer Terms
Firmness	Chewiness	Toughness
	Toughness	
	Hardness	
Smoothness	Sleek	Smooth *
	Creamy	
	Silky	
	Clean	
	Clear	
	Hard Surface	
Moistness	Wet	Juicy *
	Slimy	
	Juicy	
	Tender	
Roughness of surface	Coarse	Rough *
	Abrasive	
	Gritty	
	Jagged	
Adhesive	Sticky	Chewy
	Chewy	
	Gummy	
	Tacky	
Cohesiveness	Chewy	Gummy/Spongy **
	Gummy	
	Crumbly	
	Spongy	
	Uniform when biting down	
	Change in shape but stay as whole	
Crispiness	Crunchy	Crunchy
	Crackly	
Uniformity of bite	Consistency of texture	Consistent
	Hardness	
	Brittle	
	One bite	
	Disintegrate	
	Smooth	
	Consistent bite or consistency	
Oiliness	Oily	Oily
Astringency	Dry	Dry
	Tabasco	
	Chaps your lips	
	Bitter	
	Sour	
	Salty	
	Spicy	
	Thirsty	
	Thick	
Chew count	Chewy	Chewy
	Gummy	
Residuals in mouth	Gritty	Gritty
	Chewy (if it stuck in teeth)	
	Grainy	
Powdery	Chalky	Powdery
	Dry	
	Powdery	
Dissolvability	Disintegrate	Melts
	Airy	
	Melts	
	Dissolves	
Heat burn ^1^	Spicy	Spicy
	Hot	
	Real hot	
	Hot-hot (for Spanish foods)	
	Flaming (for cheetos)	
	Lips burning	
	Chili powder (chili sounds like cold)	
	Chili pepper (chili sounds like cold)	
Particle Amount	Crumbly	Grainy
	Grainy	
	Gritty	

^1^ Heat/burn technically is a trigeminal sensation part of flavor, separate from texture. However, it is included here because people often refer to it as part of texture because of its seemingly physical effect in the mouth. * Closest term for the product tasted but no single term because it depends on the product. ** Equal number of consumers voted for these terms.

**Table 5:** Consumer texture terms provided by the Hindi-speaking group (India).

Attributes	Consumer Terms	English Translation	Consensus Consumer Terms
Firmness	सख्त (Sakkt)	Toughness	सख्त (Sakkt)
	कड़क (Kadak)	Hard	
	ज़्यादा ज़ोर लगाने वाला (Zyada jor lagane wala)	Something that requires more power to bite	
	ज़्यादा चबाने वाला (Zyada chaabane wala)	More number of bites to eat	
Smoothness	लिस्सापन (Lissapan)	Gluey	
	चिकनापन (Chiknapan)	Smoothness/slickness	
	मृदु (Mradu)	Soft touch (closet meaning)	
	कोमल (Komal)	Soft feel	
	चिकना (Chikna)	Smooth/slippery	चिकना (Chikna)
	नरम (Naram)	Soft feel	
Moistness	पानी पानी (Pani-Pani)	Water-like	
	पिलपिला (Pilpila)	Flabby	
	भेजवाला (Bhejwala)	No English term	
	पनियाल (Paniyaal)	Water-like	
	गीला (Geela)	Wet	गीला (Geela)
Roughness of surface	कड़क (Kadak)	Hard	
	खुरदरा (Khurdara)	Rough/abrasive	खुरदरा (Khurdara)
	कुरकरा (Kurkara)	Crisp	
Adhesive	चिपचिपा (Chipchipa)	Sticky	चिपचिपा (Chipchipa)
	चिपकना (Chipakna)	Stickiness	
	चिकना (Chikna)	Smooth	
Cohesiveness	मज़बूती (Majbuti)	Strong	
	कडा (Kadaa)	Tough	
	पकड़ के रहने वाला (Pakad ke rehne wala)	Something that holds together	
	न टूटने वाला (Na tutane wala)	Something that does not break	
	न बिखरने वाला (Na bikharne wala)	Something that does not scatter	न बिखरने वाला (Na bikharne wala)
	जुड़ा हुआ (Juda hua)	Remains together	
Crispiness	कुरकुरा (Kurkura)	Crispy	कुरकुरा (Kurkura)
Uniformity of bite	हल्का (Halka)	Light	
	तोड़ने में आसान (Todne me assan)	Easy to break/bite	
	टूटने की समानता (Tutane ki samanta)	Uniformity of bite	
	चबाना आसान है (Chabana assan hai)	Easy to bite	चबाना आसान है (Chabana assan hai)
Oiliness	तेल बहुत ज्यादा है (Tael bahut jyada hai)	High amount of oil	
	तला हुआ (Tlaa) hua	Fried	
	चिकनाई (Chiknaye)	Oily	चिकनाई (Chiknaye)
Astringency	सूखापन (Sukhapan)	Dryness	सूखापन (Sukhapan)
Chew count	बहुत चबाना पड़ता है (Bahut chabana padtha hai)	Something that requires a higher number of chews to eat	बहुत चबाना पड़ता है (Bahut chabana padtha hai)
Residuals in mouth	दांतो के बीच में रह जाना (Danto ke beech me reh jana)	Stuck in between teeth	
	मुँह में रह जाता है (Muh me reh jata hai)	Leftover in mouth	मुँह में रह जाता है (Muh me reh jata hai)
Powdery	पाउडर जैसा (Powder jaisa)	Powder-like	पाउडर जैसा (Powder jaisa)
	आटे जैसा (Aatte jaisa)	Flour-like	आटे जैसा (Aatte jaisa)
Dissolvability	पिघलना (Peghalna)	Melts	पिघलना (Peghalna)
	घुलना (Ghulna)	Dissolves	घुलना (Ghulna)
Heat burn ^1^	ती्खा (Tekha)	Peppery hot	ती्खा (Tekha)
	मिर्ची वाला/मिर्ची लगी (Mirchi wala/Mirchi lagi)	Spicy	
	तेज़ (Tej)	Peppery tang	
Particle amount	मुरमुरा (Murmura)	Crisps	
	किरकिरा (Kirkira)	Gritty	
	भुरभुरा (Bhurbhura)	Crumbly	भुरभुरा (Bhurbhura)

^1^ Heat/burn technically is a trigeminal sensation part of flavor, separate from texture. However, it is included here because people often refer to it as part of texture because of its seemingly physical effect in the mouth.

**Table 6:** Consumer texture terms provided by the Spanish-speaking group.

Attributes	Consumer Terms	English Translation	Consensus Consumer Term
Firmness	Consistencia	Consistency	Resistencia
	Dureza	Hardness	
	Resistencia	Resistance	
Smoothness	Plano	Flat	Liso *(Smooth)*
	Terso	Smooth	
	Suave	Soft	
	Liso	Smooth	
	Homogéneo	Homogeneous	
Moistness	Jugosidad	Juiciness	Jugosidad
	Aguado	Watery	
	Mojado	Wet	
Roughness of surface	Rugosidad	Roughness	Aspereza
	Superficie heterogénea	Heterogeneous surface	
	Superficie Irregular	Irregular surface	
	Rasposo	Rough	
	Lijoso	Pieces with sharp edges (for example, rough surface of a nail filer)	
	Aspereza	Roughness	
Adhesive	Pegajoso	Sticky	Pegajoso
	Chicloso	Taffy	
	Gomoso	Gummy	
Cohesiveness	Elasticidad	Elasticity	Elasticidad
	Suavidad	Softness	
	Consistencia	Consistency	
	Firmeza	Firmness	
	Quebradizo	Brittle	
Crispiness	Crujencia	Crunchiness	Crujencia
	Crocancia	Crunchiness	
Uniformity of bite	Homogeneidad de la mordida	Homogeneity of bite	Homogeneidad de la mordida
	Uniformidad	Uniformity	
	Consistencia de la mordida	Consistency of bite	
	Resistencia de la mordida	Resistance of bite	
Astringency	Aspereza	Roughness	Astringencia
	Sensación de sequedad	Dryness sensation	
	Astringencia	Astringency	
Oiliness	Aceitoso/grasoso	Oily/greasy	Aceitoso/grasoso
Chew count	Masticabilidad	Chew ability	Número de masticadas
	Número de masticadas	Number of chews	
Residuals in mouth	Sabor de boca	Flavor in mouth	Residuo en boca
	Sensación de boca	Sensation in mouth	
	Residuo en boca	Residual in mouth	
Powdery	Arenoso	Sandy	Polvoroso
	Granuloso	Grainy	
	Harinoso	Floury	
	Polvoso	Dusty	
	Polvoroso	Dusty	
Dissolvability	Solubilidad	Solubility/solvability (melts)	Solubility/solvability Solubilidad
	Disolubilidad	Dissolvability	
Heat burn ^1^	Picante	Spicy	Picante
	Picosidad	Spicy (Mexican)	
	Enchiloso	Spicy (Mexican)	
Particle amount	Granuloso	Grainy	Granuloso

^1^ Heat/burn technically is a trigeminal sensation part of flavor, separate from texture. However, it is included here because people often refer to it as part of texture because of its seemingly physical effect in the mouth.

**Table 7:** Consumer texture terms provided by the Mandarin-speaking group (Chinese).

Attributes	Consumer Terms	English Meaning of Consumer Terms	Consensus Consumer Term
Firmness	有嚼劲	Chewy	有嚼劲
	韧性	Toughness	
Smoothness	圆润度	Roundness	圆润度
	圆滑	Smooth	圆滑感
	顺滑	Smooth	
Moistness	多汁	Juicy	多汁
	水润	Moist	
Roughness of surface	凹凸不平	Rugged	凹凸不平
	磨砂	Roughness	
Adhesive	黏牙	Teeth sticky	黏牙
	粘稠	Viscous	
Cohesiveness	韧性	Tenacity (something that does not break or recover in shape, like sponge)	韧性
Crispiness	脆性	Crispy	脆性
	嘎嘣	Crunchy	
Uniformity of bite	口感均匀	Even texture	口感均匀
	均匀的	Evenly	
Astringency	发涩	Dry	发涩
	麻	Numbing	
Oiliness	油腻	Greasy	油腻
	油乎乎的	Oily	
	冒油	Oily	
Chew count	嚼劲	Chewy	嚼劲
	下咽度	Easy of swallowing	
Residuals in mouth	渣	Residual	渣
Powdery	面	Powdery	面
	绵	Powdery	
	面面的	Powdery	
Dissolvability	入口即化	Dissolve directly when put in mouth	入口即化
Heat burn ^1^	烧灼	Burning	烧灼
	辣	Spicy	
	冲	Pungent	
Particle amount	碎	Granular	碎
	酥	Crisp	

^1^ Heat/burn technically is a trigeminal sensation part of flavor, separate from texture. However, it is included here because people often refer to it as part of texture because of its seemingly physical effect in the mouth.

**Table 8:** Final consumer texture terms provided by each different language consumer.

Attributes	English-Speaking Group	Spanish-Speaking Group	Hindi-Speaking Group	Mandarin-Speaking Group
Firmness	Toughness	Resistance	Toughness	Chewy
Smoothness	Smooth *	Smooth	Smooth	Roundness/smoothness **
Moistness	Juicy *	Juiciness	Wet	Juicy
Roughness of surface	Rough *	Roughness	Rough/abrasive **	Rugged
Adhesive	Chewy	Sticky	Sticky	Teeth sticky
Cohesiveness	Gummy/Spongy **	Elasticity	Something that does not scatter	Tenacity
Crispiness	Crunchy	Crunchiness	Crispy	Crispy
Uniformity of bite	Consistent	Homogeneity of bite	Easy to bite	Even texture
Astringency	Dry	Astringency	Dryness	Dry
Oiliness	Oily	Oily/greasy **	oily	Greasy
Chew count	Chewy	Number of chews	Numbers of chews	Chewy
Residuals in mouth	Gritty	Residual in mouth	Leftover in mouth	Residual
Powdery	Powdery	Dusty	Powder/flour-like **	Powdery
Dissolvability	Melts	Solubility/solvability **	Melts	Dissolve
Heat burn ^1^	Spicy	Spicy	Peppery hot	Burning
Particle amount	Grainy	Grainy	Crumbly	Granular

^1^ Heat/burn technically is a trigeminal sensation part of flavor, separate from texture. However, it is included here because people often refer to it as part of texture because of its seemingly physical effect in the mouth. * Closest term for the product tasted but no single terms because it depends on the product. ** Equal number of consumers voted for these terms.
